# The interactome of a family of potential methyltransferases in HeLa cells

**DOI:** 10.1038/s41598-019-43010-2

**Published:** 2019-04-29

**Authors:** Valentina V. Ignatova, Pascal W. T. C. Jansen, Marijke P. Baltissen, Michiel Vermeulen, Robert Schneider

**Affiliations:** 1 Institute of Functional Epigenetics, Helmholtz Zentrum München, Deutsches Forschungszentrum fuer Gesundheit und Umwelt (GmbH) Ingolstaedter Landstr. 1, 85764 Neuherberg, Germany; 2 Department of Molecular Biology, Faculty of Science, Radboud Institute for Molecular Life Sciences, Oncode Institute, Radboud University Nijmegen, Geert Grooteplein 30, 6525 GA Nijmegen, The Netherlands

**Keywords:** Methylases, Proteomics

## Abstract

Human methytransferase like proteins (METTL) are part of a large protein family characterized by the presence of binding domains for S-adenosyl methionine, a co-substrate for methylation reactions. Despite the fact that members of this protein family were shown or predicted to be DNA, RNA or protein methyltransferases, most METTL proteins are still poorly characterized. Identification of complexes in which these potential enzymes act could help to understand their function(s) and substrate specificities. Here we systematically studied interacting partners of METTL protein family members in HeLa cells using label-free quantitative mass spectrometry. We found that, surprisingly, many of the METTL proteins appear to function outside of stable complexes whereas others including METTL7B, METTL8 and METTL9 have high-confidence interaction partners. Our study is the first systematic and comprehensive overview of the interactome of METTL protein family that can provide a crucial resource for further studies of these potential novel methyltransferases.

## Introduction

The recent resurgent interest in epitranscriptomics and in particular in novel types and sites of nucleoside modifications (both on RNA and DNA) started a hunt for new nucleoside modifying enzymes. In RNA alone 171 different types of naturally occurring modifications (http://modomics.genesilico.pl/modifications/) had been identified on all types of RNAs. 72 of these modifications are different forms of methylations, however the functions of many of these methylations as well as the modifying enzymes are still unknown. Interestingly, many methylations can be removed by demethylases^1,2^. Thus, nucleoside methylation could carry more stable “epigenetic” information or could be dynamic with a high regulatory potential.

**Figure 1:**
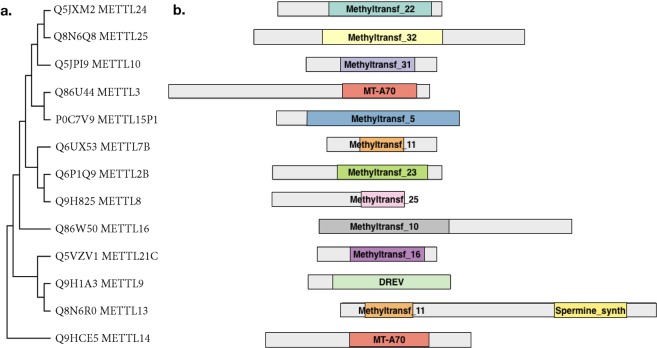
METTL family members studied. (**a**) Phylogenetic tree of METTL proteins by the Maximum Parsimony method in MEGA7. UNIPROT numbers are indicated. (**b**) METTL proteins domain organization annotated according to Pfam (Methyltransf - methyltransferase domain, MT-A70 - N6-adenosine-methyltransferase 70 kDa subunit domain, DREV - DORA reverse strand protein domain, Spermine synth - Spermine/spermidine synthase domain).

Nucleoside methyltransferases (MTase) are a diverse family of proteins, characterized by the presence of methlytranferase like domains and a structurally conserved S-adenosly methionine (SAM) binding domain that is formed by a central seven-stranded beta-sheet structure^3^ (Fig. 1). So far the DNA (cytosine-5)-methyltransferase (DNMT) protein family have been well studied and identified as DNA (Dnmt1, 3a and 3b)^4,5^, but also as RNA (Dnmt2)^6^ methyltransferases. More recently members of the methytransferase like protein family (METTL) (http://www.genenames.org/cgi-bin/genefamilies/set/963) have been shown to methylate RNA in mammals: METTL3 and METTL14 catalyze the formation of m6A (N6-methyladenosine) in mRNA^7,8^ as well as in primary-microRNAs^9^. Interestingly, to be active METTL3 has to be in a well-defined complex together with METTL14^10^. METTL1 had been shown to catalyze m7G (7-methylguanosine) formation in mRNA and tRNA in a complex with the tRNA (guanine-N(7)-)-methyltransferase non-catalytic subunit WDR4^11^. In contrast to this, another newly identified m6A RNA methyltransferase – METTL16, seems not to require additional proteins for its methyltransferases activity^12^. Additionally, three members of the METTL family were shown to be able to catalyze m3C (3-methylcytidine) in RNA^13^. Besides RNA, METTLs can also methylate other targets. METTL10^14^ and METTL11A^15^ have been shown to be protein methyltranferases, whereas it had been suggested that METTL4 might methylate DNA^16^. These recent findings highlight METTL proteins as a very interesting family of novel methyltranferases, many with potential nucleoside methyltransferase activity. However, for most METTL family members it is still unclear with which proteins they interact or whether they act by themselves. In the recent large-scale human proteome analysis only some members of METTL family were included^17^.

In order to get insights into the functions of the METTL family members we systematically tagged individual METTL proteins, purified METTL-interacting proteins from HeLa cells and identified stable interaction partners by mass spectrometry. Remarkably, whereas some METTL proteins are engaged in specific protein-protein interactions, for others we did not detect stable high-confidence interaction partners. Overall this work represents a valuable resource revealing the interactome of an important family of putative novel methyltransferases.

## Results

### Establishment of a setup to study METTL interactome

**Figure 2:**
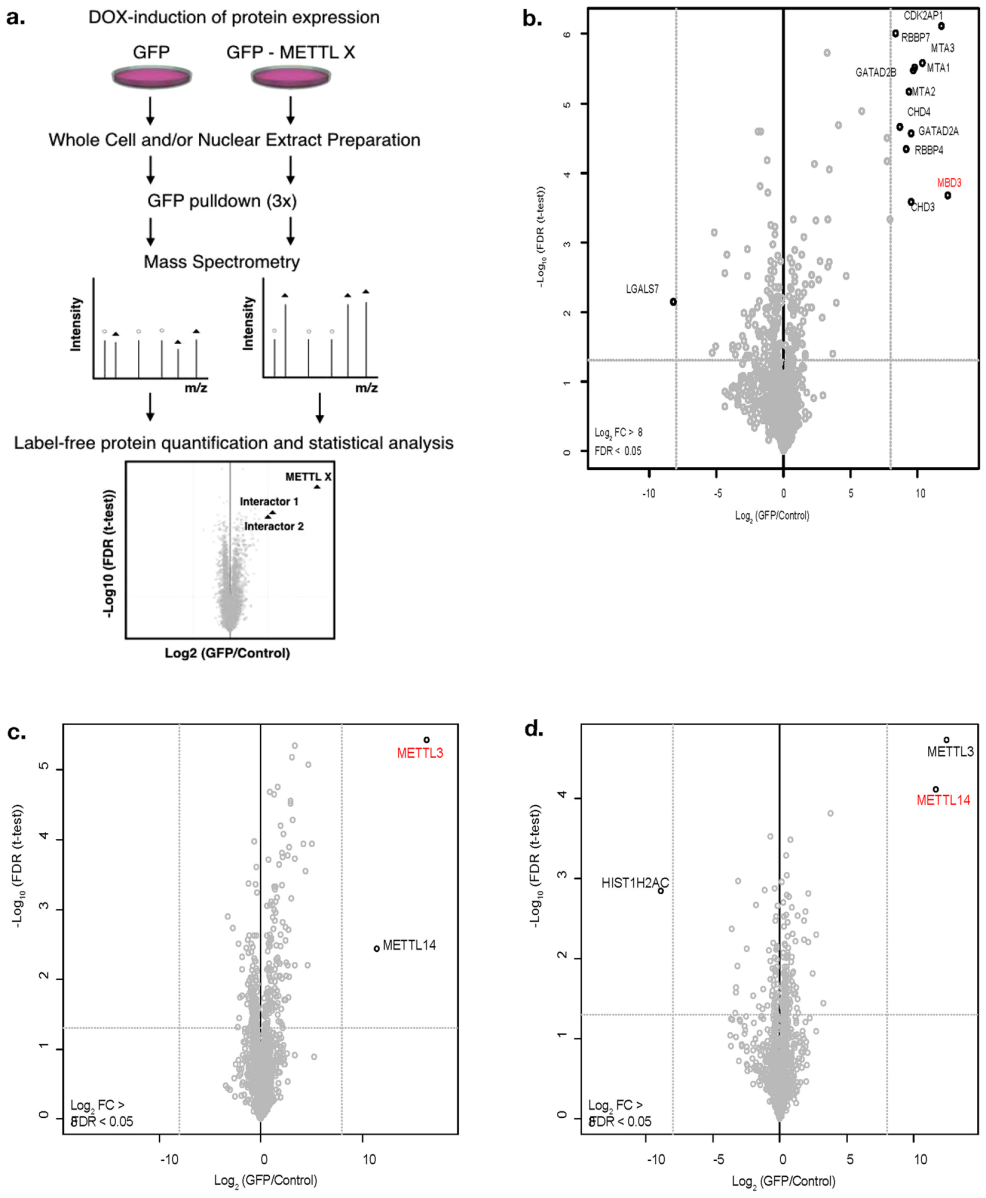
Experimental workflow. (**a**) ORFs of METTL proteins were cloned as GFP fusions under the control of a DOX-inducible promoter for targeted single copy integration into HeLa FRT cells. Whole cell extracts and/or nuclear extracts were prepared from GFP-METTL or GFP (control) expressing cells. GFP pull-downs were performed in triplicate followed by mass spectrometry analysis. Analysis of raw data was performed in MaxQuant^40^ (version 1.5.1.0) and Andromeda. Data filtering and generation of volcano plots was done essentially as described using a one-way ANOVA test^42^ with log2 fold change (FC) > 8 and false discovery rate (FDR) < 0.05 as thresholds. (**b**) Volcano plot of GFP-MBD3 interacting proteins as an example of successful complex identification using this protocol. Significant interactors are indicated. Volcano plots of (**c**) METTL3 and (**d**) METTL14 interactors. The log2 FC of GFP fusions to control in label-free quantification are plotted against the −log10 of the FDR calculated by a permutation-based FDR adapted t-test. The baits are indicated in red.

Our aim was to systematically identify interaction partners of METTL family members. Due to the absence of specific and immunoprecipitation (IP) grade antibodies for most METTL proteins, we generated N-terminal GFP-fusions of 13 METTL proteins under the control of a DOX-inducible CMV (cytomegalovirus) promoter. We used a HeLa-FRT cell line for recombination mediated targeted integration and inducible expression as described in van Nuland *et al*.^18^ (Fig. 2a). We decided to use first Methyl-CpG-binding domain protein 3 (MBD3) fused to GFP as a control to verify that under our conditions we can purify and identify well-defined protein complexes from total cellular HeLa extract by label-free quantitative mass spectrometry. As expected, we identified the majority of previously described interaction partners of MBD3 including multiple members of the Nucleosome Remodeling and Deacetylase (NuRD) complex (Fig. 2b)^19^.

### METTL interaction proteomics

**Figure 3:**
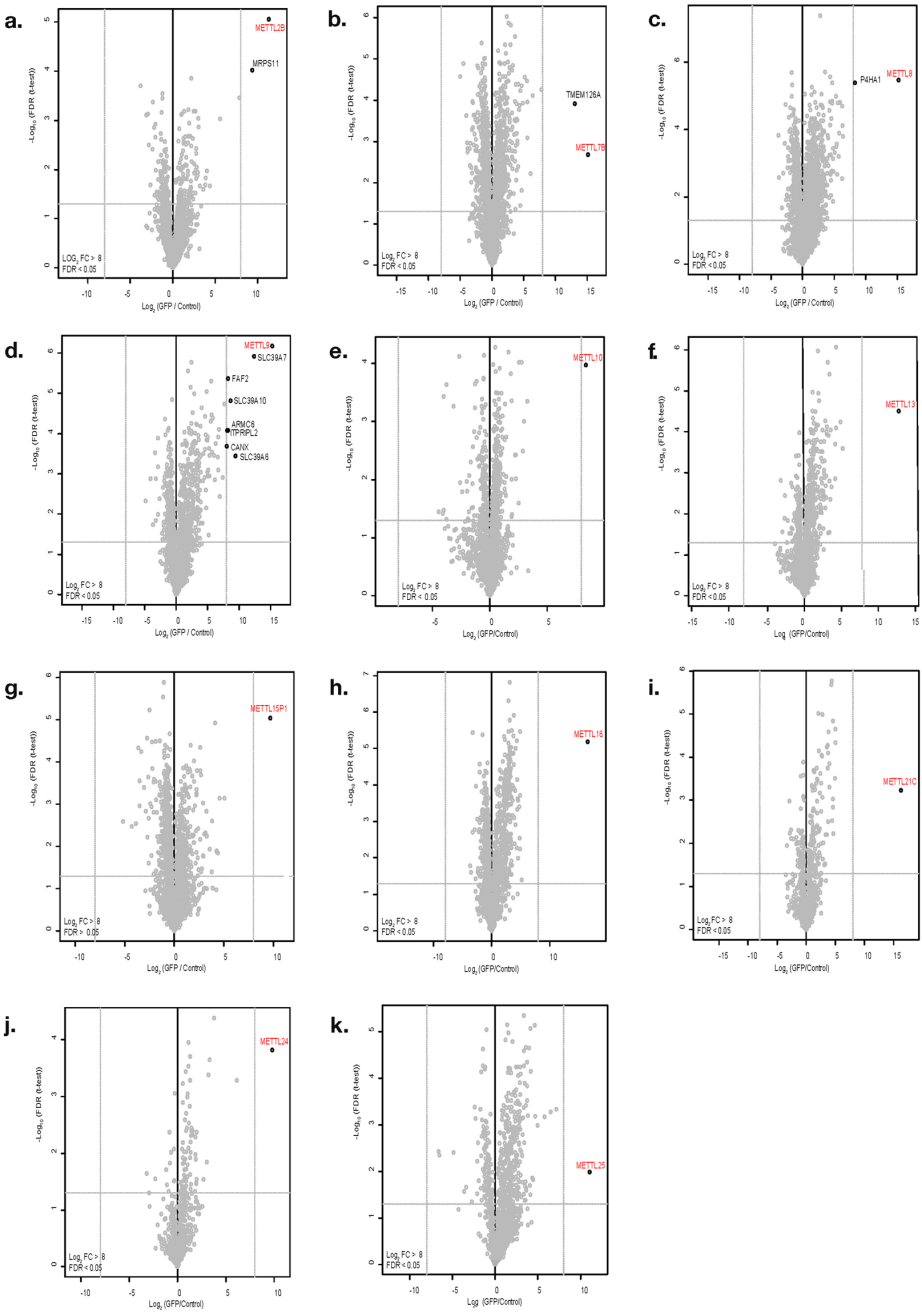
Interactome of METTL family members. Volcano plot visualization of interaction partners for: (**a**) METTL2B, (**b**) METTL7B, (**c**) METTL8, (**d**) METTL9, (**e**) METTL10, (**f**) METTL13, (**g**) METTL15P1, (**h**) METTL16, (**i**) METTL21C, (**j**) METTL24, (**k**) METTL25. Purifications were performed from whole cell extracts. Data displayed as described in the legend of Fig. 2.

We additionally tested our approach on two known methyltransferases, METTL3 and METTL14 since an interaction between METTL3 and METTL14 had been previously described^20,21^. We detected this interaction between METTL3 and METL14 both in the METTL3 purification (Fig. 2c) and in the METTL14 purification (Fig. 2d). Having validated that we can detect stable interactions we extended our studies towards 11 other members of the METTL family (Fig. 3a–k, Supplementary Table 1).

For METTL7B (Fig. 3b) we identified the transmembrane protein TMEM126A as a high confidence interactor (with a log2 fold change (FC) > 8 compared to the GFP expressing control). TMEM126A itself is a Myc proto-oncogene protein (MYC) target and a mitochondrial membrane protein of unknown function. Defects in this gene can cause optic atrophy type 7 (OPA7)^22,23^. This interaction with a mitochondrial membrane protein could suggest a potential function of METTL7B in, for instance, RNA methylation in mitochondria.

We identified prolyl 4-Hydroxylase Subunit Alpha 1 (P4HA1) as a high confidence interactor for METTL8 (Fig. 3c). P4HA1 has dioxygenase and oxidoreductase activity and catalyzes the post-translational formation of 4-hydroxyproline^24^. Its expression correlates with unfavorable prognosis in various cancers according to human protein atlas data (www.proteinatlas.org). P4HA1 was also identified as partner of number of Forkhead box proteins such as the transcription factors FOXA3, FOXG1, FOXL2, FOXP3, FOXS1 and the transcriptional activator GLI1 that can promote cancer cells migration^25^. More recently Xu *et al*.^13^ showed that METTL8 catalyzes the formation of m3C in RNA *in vitro* and in human cells. Having identified P4HA1 as an interactor for METTL8 one could speculate that METTL8 couples RNA modifications with transcriptional regulation.

Applying a threshold of log2 FC > 5 revealed additional potential interactors for METTL2B, METTL13, METTL15P1, METTL16, METTL21C, METTL24 and METTL25 (Supplementary Fig. 1a,f–k) although often close to the threshold. Surprisingly, we did not detect any interactors for METTL10 with a log2 FC > 5 (Supplementary Fig. 1e).

### METTL9 interacts with CANX

For METTL9 we identified multiple interesting interaction partners including membrane proteins such as Calnexin precursor (CANX), a potential chaperone, and multiple Solute carrier family 39 (SLC39) proteins (Fig. 3d).

**Figure 4:**
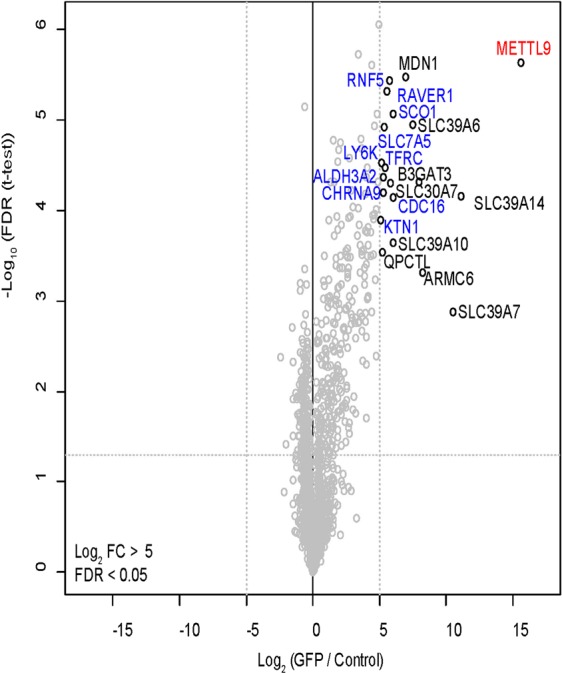
Nuclear interactome of METTL9. Volcano plot visualization of METTL9 interaction partners. Purifications were performed from nuclear extract. Data displayed as described in Fig. 2 but using cutoff log2 FC > 5. The interactors, detected only in the nuclear interactome, are indicated in blue.

Next, we repeated the purifications for METTL9 using nuclear extract (see Supplementary Fig. 2 for a control of the fractionation) instead of total cellular extract. We chose METTL9 for this experiment as an example since we found multiple interactors for this protein and wanted to specifically search for nuclear interactors. As shown in Fig. 4 we identified additional proteins interacting with METTL9 with a threshold of log2 FC > 5 (Fig. 4).

**Figure 5:**
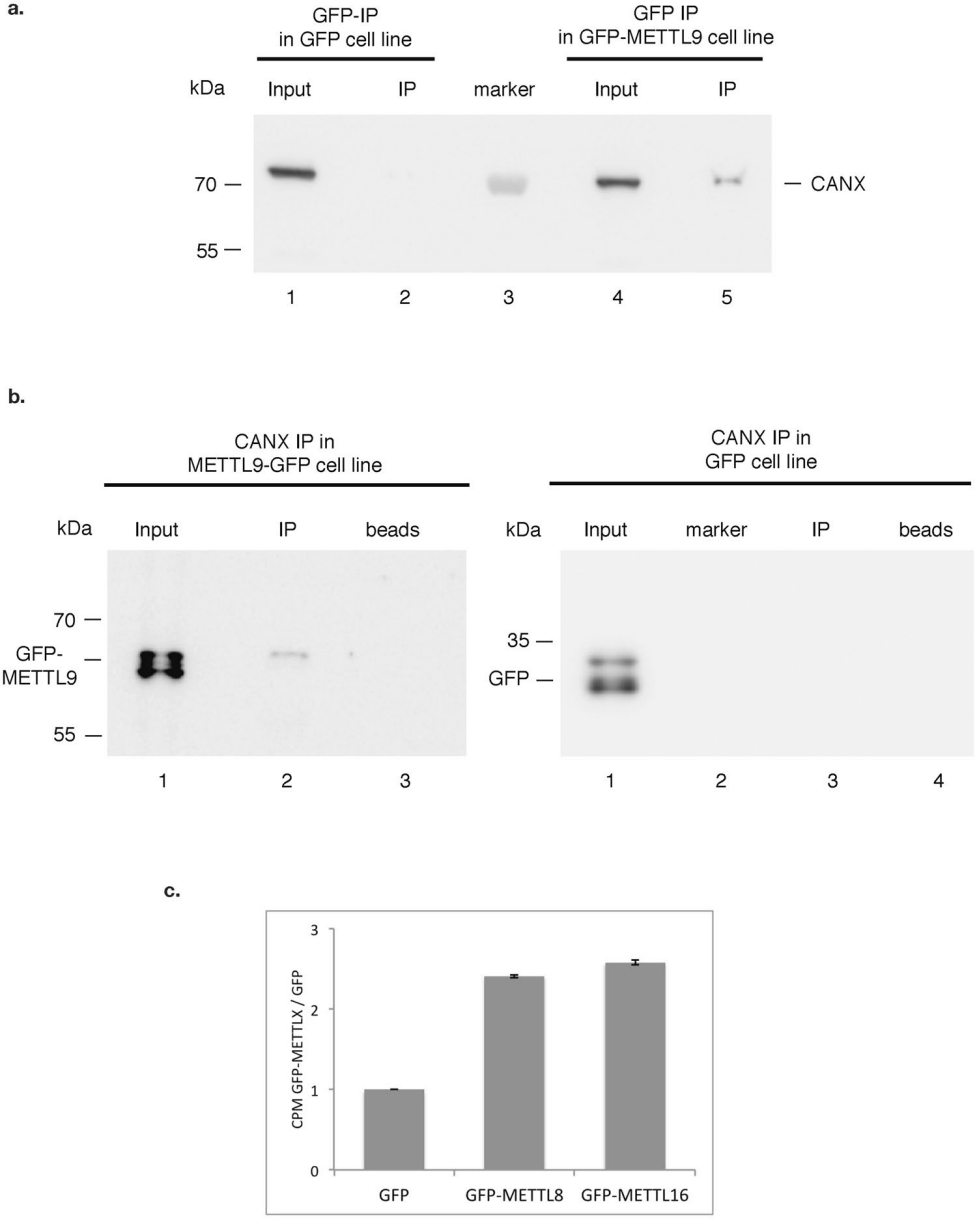
Confirmation of METTL9 interactor and enzymatic activity of GFP-METTLs. (**a**,**b**) Validation of interaction between METTL9 and CANX by co-IP. (**a**) CANX is detected in GFP-METTL9 IP (a, lane 5) but not GFP IP (a, lane 2). 10 μl of GFP trap and 2 mg of whole cell extract were used. 20 μg of Input material were loaded for a comparison. (**b**) GFP-METTL9 (left panel, line 2) but not GFP (right panel, line 3) can be detected by immuno-blot with GFP antibody in the CANX IP. 10 μg of calnexin antibody and 4 mg of whole cell extract were used. 200 μg of Input material were loaded for comparison. No antibody (beads alone) used as control. (**c**) *In vitro* methyltransferase assays demonstrating that our GFP-METTL8 and GFP-METTL16 purifications have the expected RNA methyltransferase activity. GFP (as a control) and GFP-fusion proteins were purified from corresponding DOX-induced HeLa FRT cell lines and used in an *in vitro* methyltransferase assay on total RNA from HeLa cells as a substrate and 3H-SAM as a methyl-donor. After purification of the RNA, counts per minute (CPM) were quantified by liquid scintillation counting. Ratio of CPM measured for reactions with GFP-METTL fusion proteins relative to GFP control are plotted. Data are shown as mean ± SD from three replicates.

To verify our results, we chose to confirm the interaction between METTL9 and CANX. For this we performed GFP-METTL9 immunoprecipitation and detected, as expected, CANX as an interactor by immuno blotting (Fig. 5a). We also detected GFP-METTL9 as a CANX interacting protein in the reverse IP (Fig. 5b). CANX plays an important role in the regulation of endoplasmic reticulum luminal calcium concentration^26^ and can act as a protein chaperone that assists protein folding and quality control^27^. Based on this interaction we could speculate that METTL9 might be a protein rather than an RNA methyltransferases and could couple nascent protein folding with post-translation modifications.

We wanted to confirm that with our approach we indeed enrich for previously described enzymatic activity and not e.g. loose interaction partners essential for this activity due to the presence of the GFP tag or due to our experimental procedure. For this we performed activity tests of the purifications of two enzymes (GFP-METTL8 and GFP-METTL16) that were shown to have RNA methyltransferases activity. In an *in vitro* RNA methyltransferase assay both purifications contained, as expected, methyltransferase activity towards total cellular RNA, demonstrating that we indeed do not loose essential partners required for METTLs activity (Fig. 5c).

## Discussion

METTL proteins are currently of high interest since this is a protein family considered to encompass many potential novel methyltransferases. However, for many METTL proteins it is unclear whether they are indeed active enzymes and what are their substrates: RNA, DNA or proteins. It had recently been shown that of the enzymes we investigated, in addition to METTL3^28^, also METTL16, METTL2B and METTL8 are RNA methyltransferases^12,13^. These new discoveries and the first functional characterizations of new types and sites of RNA modifications catalyzed by METTL family members^29^ has boosted the interest in this protein family in the last years.

Our aim was to get insight into the functions of METTL family members by systematically purifying and identifying their interaction partners from HeLa cells. To our knowledge this work is the first comprehensive proteomics interaction study of METTL proteins. Although we identified interesting interaction partners for METTL7B, METTL8 and METTL9, we found that many METTL members, including some that had previously been described as active methyltansferases (such as METTL16 and METTL10), seem not to have stable high confidence interaction partners (with log2 FC > 8) and thus to act without being part of well-defined complexes.

Since for many of the METTL proteins we study no commercial IP validated antibodies are available we had to use a tagging strategy. As expected, we detected all the bait proteins in our analysis (Fig. 2), but we can not fully exclude effects from the GFP tag that might e.g. affect interactions with specific partners. The advantage of our inducible expression system is that we avoid potential toxic effects or phenotypic changes of cells due to long-term over-expression of proteins that might be active enzymes. However, the overexpression of METTL proteins might affect the expression of other proteins (including other methyltransferases) and could mask interactions e.g. due to the transcriptional repression of interactors. Since we had successfully applied similar purification protocols relying on GFP fusions stably integrated in HeLa-FRT cells combined with DOX inducible expression before^17,18,30–32^ we are rather convinced that the low number of stable interaction partner we detect is not simply due to technical limitations of our approach. To additionally validate our approach we used the purification of GFP-MBD3 as a positive control and successfully identified 7 previously described interaction partners. We also successfully reproduced the previously described interaction between METTL3 and METT14, by purifying both GFP-METTL3 and GFP-METTL14. Note that the third previously reported METTL3/METT14 interacting protein – pre-mRNA-splicing regulator (WTAP) has been described to be less stably associated and therefore we did not detect it under the conditions we used^10^. Furthermore, we also showed that the N-terminal GFP tagging did not interfere with methyltransferases activity of our purifications suggesting that no interaction partners essential for METTLs activity are getting lost during our purifications. All together this strongly suggests that our approach works technically and that many METTLs indeed might act outside of stable complexes and might not need partners to be active. Interestingly, multiple METTLs have potential substrate binding ability e.g. for RNA^33–35^ that could allow them to bind their substrates and thus act alone. In line with this, a recently published crystal structure of METTL16 bound to its target RNA^36^ revealed the structural basis for RNA binding and methylation by METTL16 alone and supports our conclusion that many METTL proteins may act outside of stable protein complexes. This would be comparable to many kinases that are important enzymes and often lack strong interactors. However, we want to point out that this does not exclude additional transient weaker interactions that we did not identify in our affinity purifications-mass spectrometry experiments. For this, alternative approaches such as cross-linking mass spectrometry or proximity labeling such as APEX^37^ or bioID^38^ could be pursued to capture enzyme-substrate relations. Furthermore, it is possible that potential interactions might not be present in the HeLa cell line we used.

Our work systematically characterized for the first time the interactome of an important family of putative novel methyltransferases. It will be a crucial resource and starting point for future studies on the functional significance of the interactions that we report. Since there is currently a lack of high quality antibodies for many METTL proteins, the large set of cell lines we created, expressing inducible GFP fusions of METTL proteins, could be a valuable tool for further studies such as identifications of the potential binding sites of these proteins to RNAs or DNAs as well as to screen for potential substrates and modification sites.

## Methods

### Plasmids, cell culture and cell lysis

Open reading frames (ORFs) of the METTL proteins were obtained from OriGene. The DNA sequences of ORFs were amplified and cloned into a GATEWAY-compatible plasmid, pcDNA5/FRT/TO containing green fluorescent protein (GFP)^18^. The proteins were tagged with GFP at the N terminus. Stable GFP-METTLs doxycycline inducible (DOX) cell lines were created by transfecting HeLa-FRT cells^18^ with modified pcDNA5/FRT/TO and pOG44 plasmids. HeLa-FRT cells inducibly expressing GFP-METTL constructs were cultured in high glucose Dulbecco’s modified Eagle medium (DMEM) supplemented with 10% fetal bovine serum and 1% penicillin–streptomycin (Life Technologies, Inc.). Cells, at a confluency of 80%, were treated with doxycycline at a final concentration of 1 μg/ml for 16 h to induce expression of the GFP fusion proteins. Cells were then harvested, extensively washed with PBS and lysed by adding 5 cell pellet volumes of lysis buffer (1% NP40, 150 mM NaCl, 50 mM Tris pH 8.0, 10% Glycerol, 0.5 mM DTT and 1X Complete Protease Inhibitors (Roche)). Cells were vortexed for 30 s and then incubated for 1.5 h at 4 °C on a rotation wheel. Samples were then transferred to 2 ml Eppendorf tubes and centrifuged at 21000 × g for 15 min, after which soluble whole cell extracts (WCEs) were aliquoted and snap frozen until further usage. The protein concentration of the lysates was determined using the Bradford assay.

### Nuclear and cytoplasmic extract preparation

Cells were harvested, extensively washed with PBS and resuspended in 5 volumes of a buffer containing 10 mM Hepes KOH pH 7.9, 1.5 mM MgCl2, 10 mM KCl followed by incubated for 10 minutes on ice. After centrifugation, cells were resispended in 2 volumes of a buffer containing 10 mM Hepes KOH pH 7.9, 1.5 mM MgCl2, 10 mM KCl supplemented with 1X Complete Protease Inhibitors (Roche) and 0.15% NP40 and dounced with 30–40 strokes with a type B pestle (tight). 10 seconds break was taken per 10x dounces. Suspension was centrifuged at 3200 × g for 15 minutes. The supernatant was collected as the cytoplasmic extract.

Pellet of the crude nuclei was washed with PBS and centrifuged at 3200 g for 5 minutes, resuspended in 2 volumes of a buffer containing 420 mM NaCl, 20 mM Hepes KOH pH 7.9, 20% v/v glycerol, 2 mM MgCl2, 0.2 mM EDTA supplemented with 1X Complete Protease Inhibitors (Roche), 0.1% NP40 and 0.5 mM DTT. Tubes with the suspension were rotated for an hour in the cold room. Both nuclear suspension and the cytoplasmic extract were centrifuged at maximum g for 30 min in a table top centrifuge. Extracts were aliquoted and snap-frozen in liquid N2 until further use.

Purity of nuclear and cytoplasmic fractions was confirmed by Immunoblot. 25 μg of nuclear extract or cytoplasmic extract mixed with 4x SDS loading dye was separated on a 12% SDS-PAGE. SDS-PAGE gels were blotted onto nitrocellulose membrane with the Trans-Blot Turbo transfer system (Bio-Rad). Blots were blocked in 5% skimmed milk in TBS-T. Primary antibodies against nuclear marker HDAC1 (sc-7872, Santa Cruz Biotechnology) and cytoplasmic marker Tubulin (T6074, Sigma-Aldrich) were used. Polyclonal Swine Anti-Rabbit Immunoglobulins/HRP (P0399, Dako) and Polyclonal Rabbit Anti-Mouse Immunoglobulins/HRP (P0260, Dako) were used as secondary antibodies respectively. Protein bands were visualized using Super Signal West Pico PLUS Chemiluminescent Substrate (34580, Thermo) according to the manufacturers protocol.

### GFP affinity purification

For each label free affinity purification, 3 mg of WCE or NE was incubated with 7,5 μl GFP nanobody sepharose beads (Chromotek) in a total volume of 600 μl RIPA buffer (150 mM NaCL, 50 mM Tris-HCl pH 8.0, 0.5 mM DTT and 1X Complete Protease Inhibitor cocktail) supplemented with 50 μg/ml ethidium bromide for 1.5 h at 4 °C on a rotation wheel. Pull-downs from GFP-METTL, GFP and WT cells were performed in triplicate. After incubation, beads were washed twice with 1 ml of RIPA buffer containing 300 mM NaCl, twice with 1 ml PBS with 1% NP40 and finally three times with 1 ml PBS. After the last wash, all liquid was carefully removed from the beads using a 30 G syringe. GFP-METTL, GFP and WT control samples were prepared on the same day and analysed by LC-MS/MS sequentially.

### On-bead digestion and LC-MS/MS analyses

Purified proteins on beads were denatured and eluted in elution buffer (2 M Urea, 100 mM Tris/HCL pH8.0 and 10 mM DTT) for 20 min at RT in a shaking incubator (1000 rpm). Iodoacetamide was added to a final concentration of 50 mM, and samples were then incubated in the dark for 10 min. 0.35 μg of trypsin (Promega) was then added and samples were digested overnight at RT. Digested peptides were acidified with 10% TFA and then desalted and stored on C18 StageTips^39^ prior to mass spectrometry analyses. Samples were measured on an Easy-nLC1000 (Thermo) connected online to an LTQ-Orbitrap-Fusion mass spectrometer from Thermo, using a 114 min gradient of acetonitrile (7–32%) followed by brief washes at 50% and then 90% acetonitrile, for 140 min of total data collection. Scans were recorded in data-dependent top-speed mode of a 3-s cycle with dynamic exclusion set at 60 s. Peptides were searched against the curated UniProt human proteome database (release December 2015) with MaxQuant^40^ (version 1.5.1.0) and its integrated search engine Andromeda^41^. Cysteine carbamidomethyl was used as a fixed modification, and N-terminal acetylation and methionine oxidation were used as variable modifications. Mass tolerance for precursor ions was set to 20 ppm, and mass tolerance for fragment ions was set to 0.5 Da. Additional options Match between runs, LFQ and iBAQ were also selected. Data filtering and generation of volcano plots was done essentially as described using a one-way ANOVA test^42^. The threshold for significant interactors is based on both the FDR and the ratio between GFP-METTLs and control sample. As a threshold to identify high confidence interactors we used FDR < 0.05 and FC > 8.

### *In vitro* methyltransferase assay

GFP, GFP-METTL8 and GFP-METTL16 were purified from stable HeLa FRT cell lines using GFP nanobody sepharose beads (Chromotek). On beads methyltransfease assays were performed in 6 mM HEPES-KOH (pH 8), 0.4 mM EDTA, 10 mM DTT, 80 mM KCl, 1.5 mM MgCl2, 0.2 U/mL RNasin, 1.6% glycerol, in the presence of 460 nM [3 H]-SAM (Perkin Elmer). 5 μg of total RNA from HeLa cells per reaction was used as a substrate. Assays were performed at RT o/n, followed by acid phenol chloroform extraction and column purification (Zymo Research). Tritium incorporation was analyzed by liquid scintillation counting using Triathler counter (HIDEX) in Ultima Gold LSC-cocktail (Perkin Elmer) and shown as counts per minute (CPM). All data of *in vitro* methyltransferase assays are shown as mean ± SD (standard deviation) from three replicates.

### Co-immunoprecipitation (co-IP) and Western blot

Whole cell extracts were prepared as described above. Total protein concentration was measured with Pierce™ BCA Protein Assay Kit (Thermo Fisher Scientific). 4 mg of whole cell extract was used per immunoprecipitation (IP). IP with GFP nanobody sepharose beads (Chromotek) was performed as described above. For Calnexin-IP, 10 μg of anti-Calnexin antibody from Abcam per IP was used. Input and IP samples were separated by SDS–PAGE and transferred to nitrocellulose membrane, followed by immunostaining. The antibodies used for immunostaining: anti-GFP (1:50; HMGU monoclonal antibody facility) or anti-Calnexin (1:1000; ab22595, Abcam) primary antibodies followed by horseradish peroxidase (HRP)-conjugated secondary antibodies goat anti-mouse (1:1500, P0447, Dako) and anti-rabbit (1:100000, 111-035-003, Jackson Laboratory) correspondingly.

## Supplementary information

Supplementary materials

Dataset 1

## Data Availability

The proteomics data have been deposited to the ProteomeXchange Consortium via the PRIDE^43^ partner repository with the dataset identifier PXD011125.
