# Supplementary material for: The interactome of a family of potential methyltransferases in HeLa cells
Source: Sci Rep. 2019 Apr 29;9:6584. doi: 10.1038/s41598-019-43010-2 (PMC6488577; doi:10.1038/s41598-019-43010-2)
Supplement: Supplementary file 1 — Supplementary materials [file 41598_2019_43010_MOESM1_ESM.pdf]

The interactome of a family of potential methyltransferases in HeLa cells

Valentina V Ignatova[1], Pascal W.T.C Jansen[2], Marijke P Baltissen[2], Michiel Vermeulen[2★], Robert Schneider[1★]

[1]Institute of Functional Epigenetics, Helmholtz Zentrum München, Deutsches Forschungszentrum fuer Gesundheit und Umwelt (GmbH) Ingolstaedter Landstr. 1, 85764, Neuherberg, Germany,

[2] Department of Molecular Biology, Faculty of Science, Radboud Institute for Molecular Life Sciences, Oncode Institute, Radboud University Nijmegen, Geert Grooteplein 30, 6525 GA Nijmegen, The Netherlands,

★Corresponding authors.

List of supplementary material

Supplementary Figure 1. Interactome of METTL family members (log2 fold change >5).

Supplementary Figure 2. Immuno-blot of nuclear and cytoplasmic fractions.

Supplementary Figure 3. Uncropped full size immuno-blot used for Figure 5a.

Supplementary Figure 4. Uncropped full size immuno-blot used for Figure 5b.

Supplementary Table 1. Summary of METTL-interacting proteins.

Supplementary Dataset 1. Excel table with protein and peptide identification data.

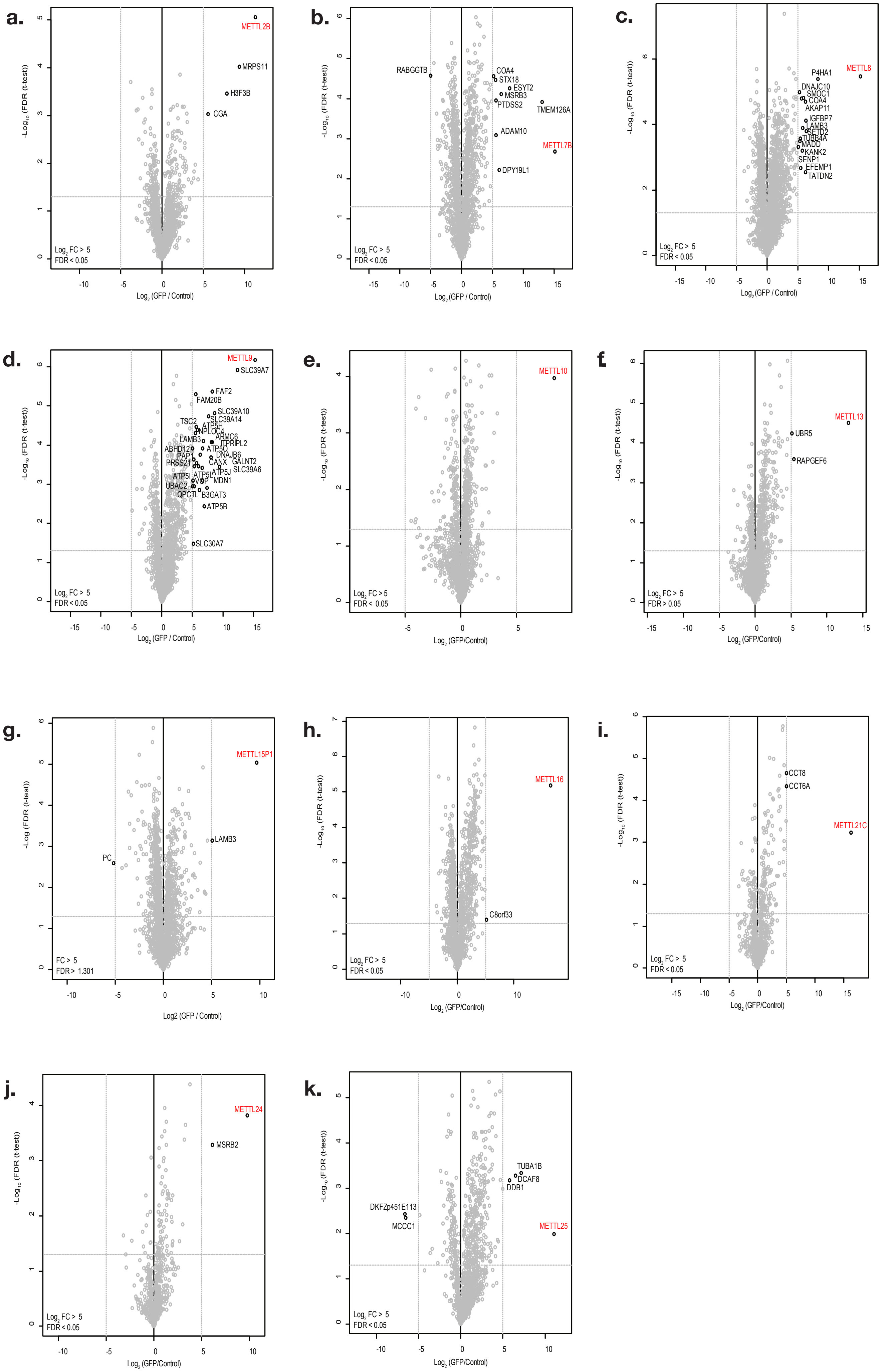
a.
METTL2B
MRPS11
H3F3B
CGA
Log2 FC > 5
FDR < 0.05
-Log10 (FDR (t-test))
Log2 (GFP / Control)
b.
RABGGTB
COA4
STX18
ESYT2
MSRB3
PTDSS2
TMEM126A
ADAM10
METTL7B
DPY19L1
Log2 FC > 5
FDR < 0.05
-Log10 (FDR (t-test))
Log2 (GFP / Control)
c.
P4HA1
METTL8
DNAJC10
SMOC1
COA4
AKAP11
IGFBP7
LAMB3
SETD2
TUBB4A
MADD
KANK2
SENP1
EFEMP1
TATDN2
Log2 FC > 5
FDR < 0.05
-Log10 (FDR (t-test))
Log2 (GFP / Control)
d.
METTL9
SLC39A7
FAF2
FAM20B
SLC39A10
SLC39A14
TSC2
ATP5H
NPLOC4
ARMC6
LAMB3
ITPRIPL2
ABHD12
ATP5O
PAP1
DNAJB6
PRSS21
CANX
GALNT2
ATP5J
SLC39A6
ATP5I
ATP5L
UBAC2
VCP
MDN1
QPCTL
B3GAT3
ATP5B
SLC30A7
Log2 FC > 5
FDR < 0.05
-Log10 (FDR (t-test))
Log2 (GFP / Control)
e.
METTL10
Log2 FC > 5
FDR < 0.05
-Log10 (FDR (t-test))
Log2 (GFP/Control)
f.
METTL13
UBR5
RAPGEF6
Log2 FC > 5
FDR > 0.05
-Log10 (FDR (t-test))
Log2 (GFP/Control)
g.
METTL15P1
LAMB3
PC
FC > 5
FDR > 1.301
-Log (FDR (t-test))
Log2 (GFP / Control)
h.
METTL16
C8orf33
Log2 FC > 5
FDR < 0.05
-Log10 (FDR (t-test))
Log2 (GFP/Control)
i.
CCT8
CCT6A
METTL21C
Log2 FC > 5
FDR < 0.05
-Log10 (FDR (t-test))
Log2 (GFP/Control)
j.
METTL24
MSRB2
Log2 FC > 5
FDR < 0.05
-Log10 (FDR (t-test))
Log2 (GFP/Control)
k.
TUBA1B
DCAF8
DDB1
DKFZp451E113
MCCC1
METTL25
Log2 FC > 5
FDR < 0.05
-Log10 (FDR (t-test))
Log2 (GFP/Control)

Supplementary Figure 1. Interactome of METTL family members (log2 fold change >5). Volcano plot visualization of interaction partners for: (a) METTL2B, (b) METTL7B, (c) METTL8, (d) METTL9, (e) METTL10, (f) METTL13, (g) METTL15P1, (h)) METTL16, (i) METTL21C, (j) METTL24, (k) METTL25. Purifications were performed from whole cell extract. Data displayed as described in the legend of Figure 2 using log2 fold change (FC) > 5 and false discovery rate (FDR) < 0.05 as thresholds. The baits are indicated in red.

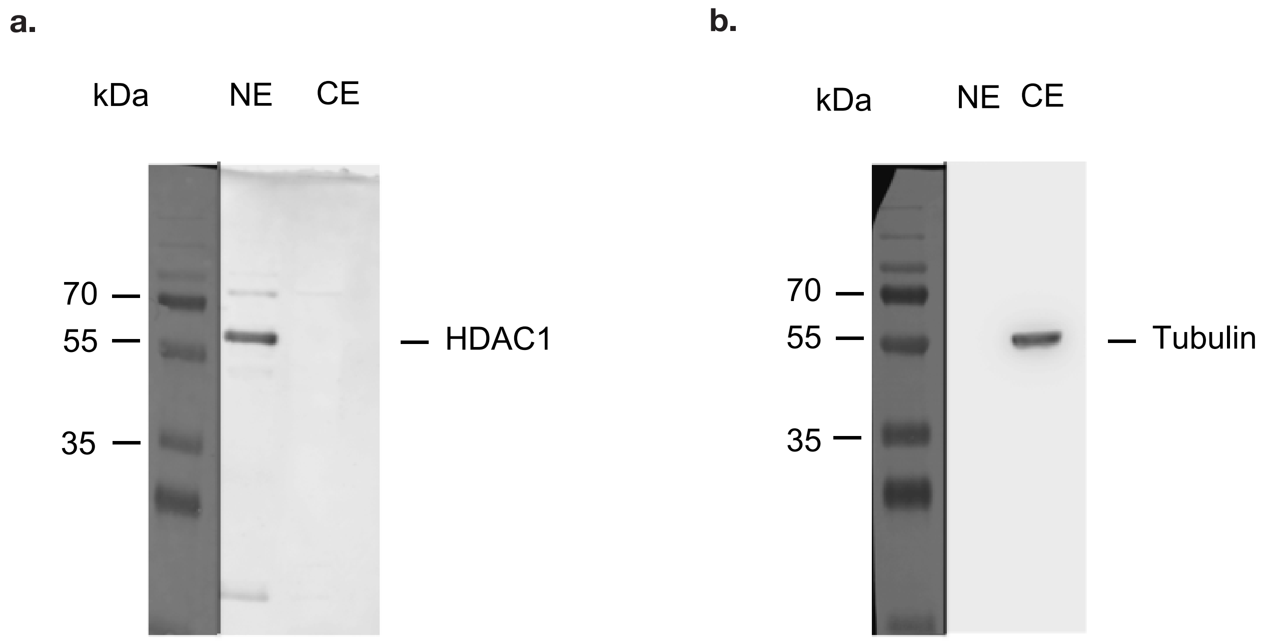

Supplementary Figure 2. Immuno-blot of nuclear and cytoplasmic fractions. HDAC1 (a) was detected in nuclear extract (NE) but not in cytoplasmic extract (CE). Tubulin (b) was detected in cytoplasmic but not in nuclear extract. Extract were probed with the indicated antibodies as markers for successful fractionation.

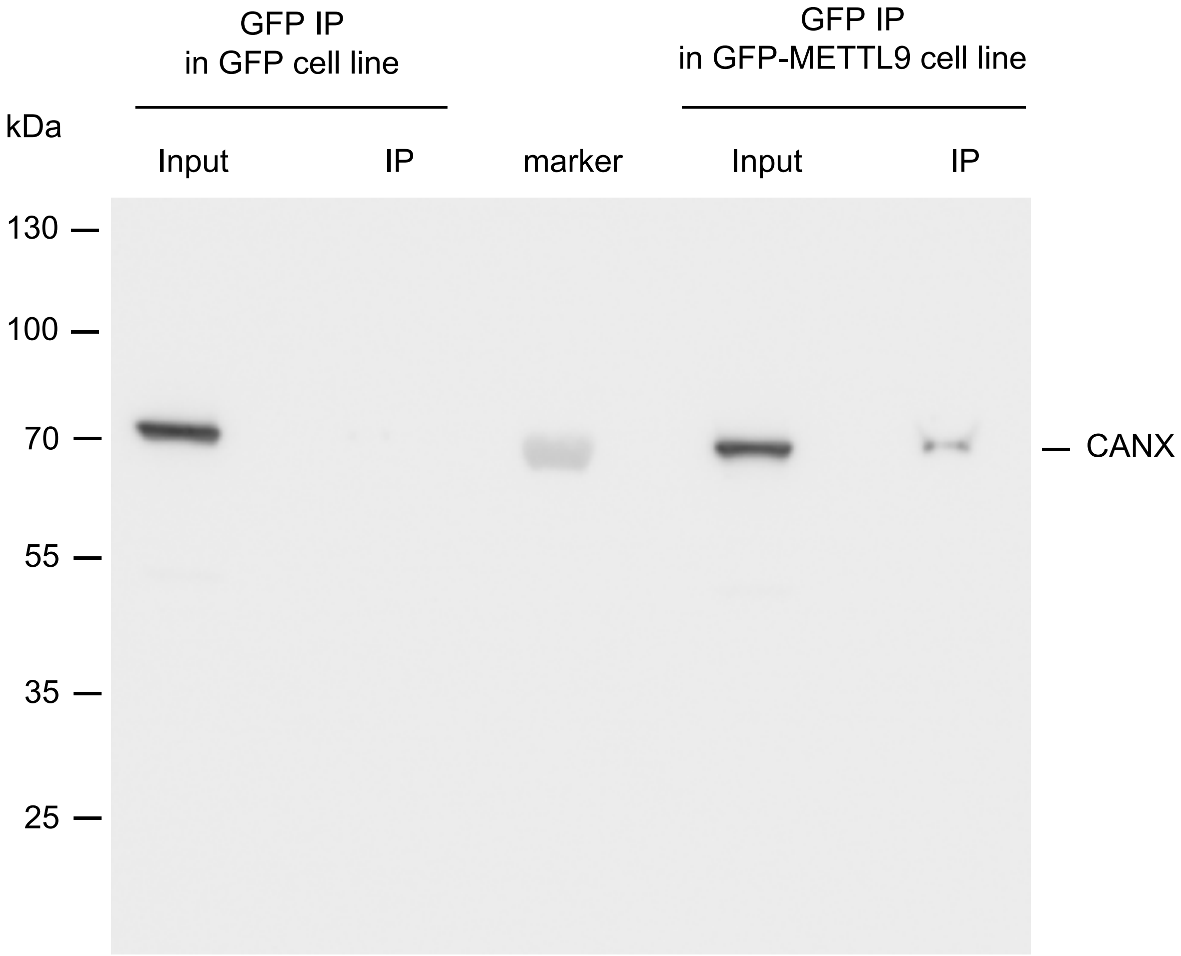

Supplementary Figure 3. Uncropped full size immuno-blot used for Figure 5a.

Validation of interaction between METTL9 and calnexin by co-IP. CANX can be detected in GFP-METTL9 IP but not in GFP IP. Labelling corresponds to Fig. 5a.

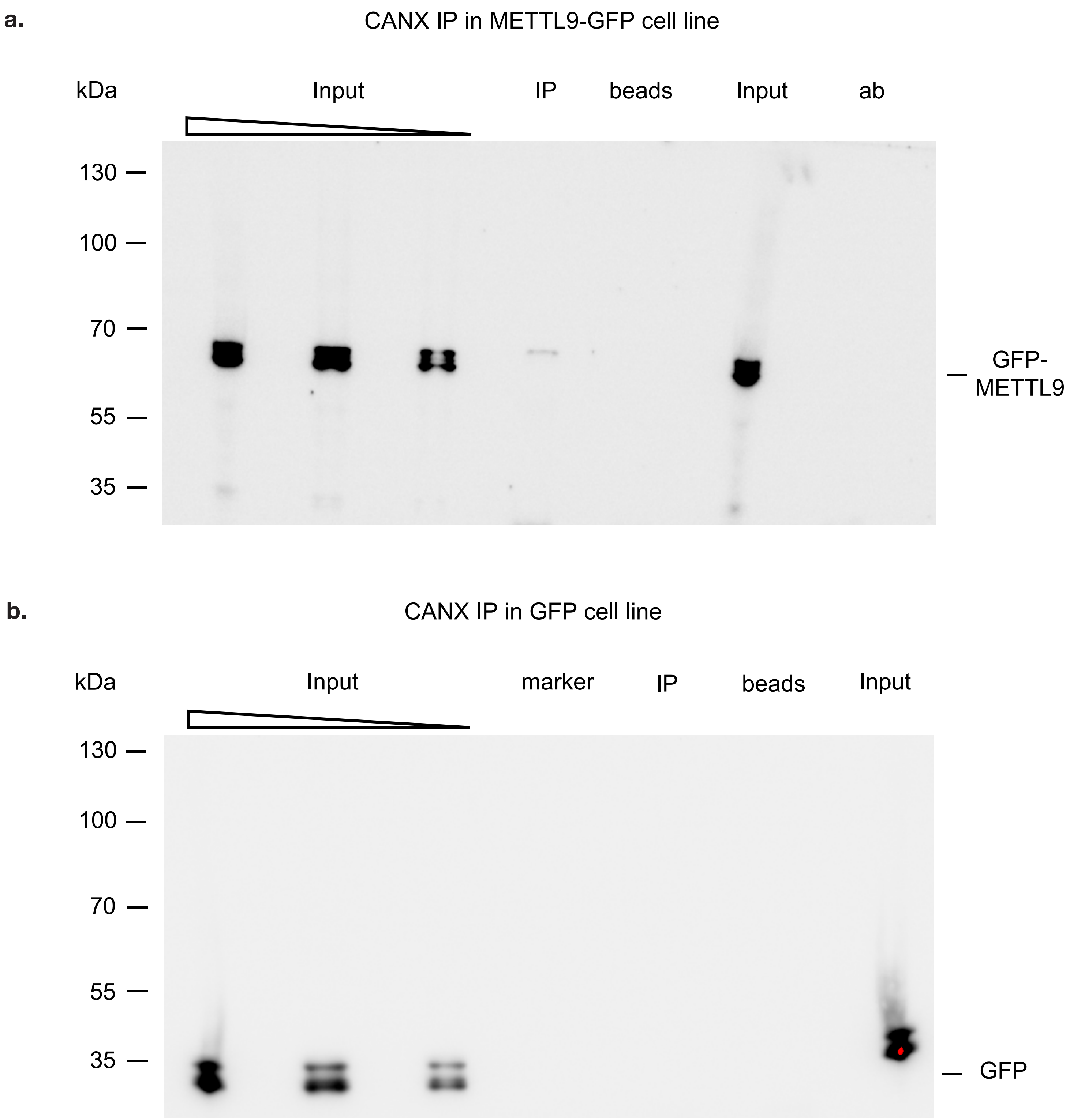

Supplementary Figure 3. Uncropped full size immuno-blots used for Figure 5b. Validation of interaction between METTL9 and Calnexin by co-IP. GFP-METTL9 (a) but not GFP (b) is detected in IP with CANX antibody. Labelling corresponds to Fig. 5b left and Fig. 5b right, correspondingly. Beads indicates no antibody control, ab indicates CANX antibody-only control. To generate Fig. 5b immuno-blots were cropped to encompass only lanes with the lowest amount of Input (200 μg), IP, beads and, for the Fig. 5b right, marker.

Table 1. Summary of METTL-interacting proteins.

Bait	Interacting protein	Function
METTL2B	MRPS11	28S ribosomal protein S11, mitochondrial
METTL7B	TMEM126A	Transmembrane protein 126A, mitochondrial
METTL8	P4HA1	Prolyl 4-hydroxylase subunit alpha-1. Catalyzes the post-translational formation of 4-hydroxyproline in -Xaa-Pro-Gly- sequences
METTL9	SLC39A7	Zinc transporter SLC39A7
	SLC39A6	Zinc transporter ZIP6
	SLC39A10	Zinc transporter ZIP10
	CANX	Calcium-binding protein, Chaperone. Potential role in a quality control of incorrectly folded proteins
	FAF2	FAS-associated factor 2. Potential role in unfolded protein response, lipid droplet degradation, insulin-like growth factor receptor signaling
	ARMC6	Armadillo repeat- containing protein 6 pathway
	ITPRIPL2	Inositol 1,4,5-trisphosphate receptor-interacting protein-like 2

Proteins were selected using as a cutoff log2 FC > 8. The functions of interacators according to Uniprot (www.uniprot.org) are indicated functions are indicated.
